# Metabolism and signaling crosstalk in glioblastoma progression and therapy resistance

**DOI:** 10.1002/1878-0261.13571

**Published:** 2023-12-26

**Authors:** Laura Zarzuela, Raúl V. Durán, Mercedes Tomé

**Affiliations:** ^1^ Centro Andaluz de Biología Molecular y Medicina Regenerativa – CABIMER, Consejo Superior de Investigaciones Científicas Universidad de Sevilla, Universidad Pablo de Olavide Seville Spain

**Keywords:** cell signaling, glioblastoma, metabolism, temozolomide, therapy resistance

## Abstract

Glioblastoma is the most common form of primary malignant brain tumor in adults and one of the most lethal human cancers, with high recurrence and therapy resistance. Glioblastoma cells display extensive genetic and cellular heterogeneity, which precludes a unique and common therapeutic approach. The standard of care in glioblastoma patients includes surgery followed by radiotherapy plus concomitant temozolomide. As in many other cancers, cell signaling is deeply affected due to mutations or alterations in the so‐called molecular drivers. Moreover, glioblastoma cells undergo metabolic adaptations to meet the new demands in terms of energy and building blocks, with an increasing amount of evidence connecting metabolic transformation and cell signaling deregulation in this type of aggressive brain tumor. In this review, we summarize some of the most common alterations both in cell signaling and metabolism in glioblastoma, presenting an integrative discussion about how they contribute to therapy resistance. Furthermore, this review aims at providing a comprehensive overview of the state‐of‐the‐art of therapeutic approaches and clinical trials exploiting signaling and metabolism in glioblastoma.

## Graphical abstract

Glioblastoma is one of the most lethal human cancers with no effective treatment nowadays. Here, we review the increasing amount of evidence supporting the connection between cell signaling deregulation and metabolic reprogramming as a key event in glioblastoma resistance to cancer therapy. Novel therapeutic approaches and current clinical trials targeting signaling and metabolism in glioblastoma are also reviewed.

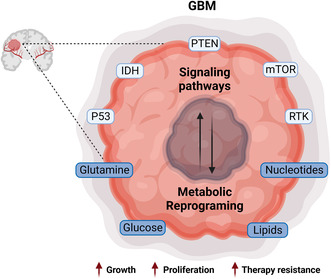

**Abbreviations**
ACBP: acyl‐CoA‐binding protein
ACC: acetyl‐CoA carboxylase
ACSS2: acetyl‐CoA synthetase enzyme
CPT1A: carnitine palmitoyltransferase 1A
DHODH: dihydroorotate dehydrogenase
FAO: fatty acids oxidation
FASN: fatty acid synthase
GBM: glioblastoma
GDH: glutamate dehydrogenase
GFAT1: glutamine‐fructose‐6‐phosphate transaminase
GLS: glutaminase
GLUT: glucose transporter
GS: glutamine synthetase
GSCs: glioblastoma stem cells
GSI: γ‐secretase inhibitors
HIF1α: hypoxia inducible factor‐1α
HK2: hexokinase 2
IDH: isocitrate dehydrogenase gene
LD: lipid droplets
LDHA: lactate dehydrogenase A
MGMT: *O*‐6‐methylguanine‐DNA methyltransferase
mTOR: mammalian target of rapamycin
NAC: *N*‐acetylcysteine
NAMPT: nicotinamide phosphoribosyltransferase
OS: overall survival
OXPHOS: oxidative phosphorylation
PGE2: prostaglandin E2
RET: reverse electron transfer
ROS: reactive oxygen species
RTK: receptor tyrosine kinase
S6K: ribosomal protein S6 kinase 1
SREBP1: sterol regulatory element‐binding protein‐1
SVZ: subventricular zone
T‐ALL: T‐cell acute lymphoblastic leukemia
TCA: tricarboxylic acid cycle
TMZ: temozolomide
WHO: World Health Organization
αKG: α‐ketoglutarate

## 1 Glioblastoma as an unmet need

Glioma is the most common form of intracranial tumor and represents approximately 80.8% of malignant brain tumors, of which 57.7% belong to the most aggressive type, the glioblastoma (GBM). GBM is the most frequent brain tumor in adults with an incidence of 3.23 per 100 000 population [1]. The median age at diagnosis is 65 years and the incidence is 1.59 times higher in males compared to females. Despite the scientific advances, GBM is still one of the most lethal human cancers due to a high recurrence and therapy resistance [1, 2]. The median survival time is 8–14 months (regardless of treatment) and the 5‐year survival rate ranges between 20% in young adults and 5% in adults over 55 years [1]. GBMs display extensive genetic and cellular heterogeneity which makes it difficult to establish a common therapeutic approach. GBM intratumoral heterogeneity is considered a key determinant of treatment failure and disease recurrence. It is manifested at the genetic, transcriptional, metabolic, and functional levels [3]. Single‐cell RNA sequencing analysis has demonstrated heterogeneous transcriptome profiles in primary GBM, including transcriptional programs related to oncogenic signaling, proliferation and hypoxia among others [4, 5, 6]. Epigenetic characterization has provided evidence of heterogeneity regarding DNA methylation within GBM, even showing spatio‐longitudinal heterogeneity [7]. Moreover, single cell‐derived clones exhibit functional heterogeneity regarding tumorigenicity, drug response or proliferation [4]. Glioblastoma stem cells (GSCs) are neural stem‐like cells identified as the GBM initiating cells. Indeed, GSCs are one of the main sources of heterogeneity with stem cell properties characterized by high entropy, self‐renewal and differentiation capabilities [8, 9]. This GSC state is the result of a permissive epigenetic landscape which allows transcriptional fluctuations and the diversity of the population [10]. In this direction, Dirkse et al. [11] propose that the heterogeneity observed in GSC‐associated cell membrane markers is due to the plasticity of cancer cells to respond to microenvironment cues with a dynamic process of reversible state transition. This heterogeneity and the ability to evade or adapt to therapy make it clear there is a need for more personalized strategies based on the biology and particularities of each GBM.

### 1.1 Clinical classification

The 2021 World Health Organization (WHO) classification of Tumors of the Central Nervous System has included new tumor types and subtypes based on the development of recent molecular parameters employed as biomarkers [12]. The current WHO classification for gliomas consists of four groups: adult‐type diffuse glioma; pediatric‐type diffuse low‐grade glioma; pediatric‐type diffuse high‐grade glioma; and circumscribed astrocytic glioma, each of them with several subtypes [12] (Table 1). GBM is grouped within the adult‐type diffuse glioma that arises *de novo* as a grade 4. Secondary GBM tumors, developing from a lower grade astrocytoma and generally with better prognosis than *de novo* (primary) GBM [13], are currently classified as grade 4 astrocytoma with mutation in the isocitrate dehydrogenase gene (*IDH*) (Table 1). GBM classification is changing continuously, attending to the molecular markers described along the scientific and technological advances. The first classifications were carried out in 1990, when scientists identified a variety of molecular markers from techniques like PCR and allele analysis [14]. Several classifications followed this one, highlighting the transcriptomic‐based molecular subtypes established in 2010 by Verhaak et al. [15] (proneural, neural, classical and mesenchymal). Later studies demonstrated the neural subtype as a neural lineage contamination which led to a reclassification that includes only the proneural, classical, and mesenchymal subtypes [16]. An additional classification based on a hierarchical consensus clustering of the DNA methylation status of primary GBM samples proposes six GBM methylation clusters (M1–M6) [17]. Correlation between methylation clusters and molecular subclasses was identified in this study, with cluster M1 enriched for the mesenchymal subtype and cluster M3 enriched for the classical subtype.

**Table 1:** Glioma classification according to 2021 WHO Classification of Tumors of the Central Nervous System [12].

Glioma type	Grade
Adult‐type diffuse gliomas	
Astrocytoma, IDH‐mutant	2, 3, 4
Oligodendroglioma, IDH‐mutant, and 1p/19q‐codeleted	2, 3
Glioblastoma, IDH‐wild type	4
Pediatric‐type diffuse low‐grade gliomas	
Diffuse astrocytoma, MYB‐ or MYBL1‐altered	1
Angiocentric glioma	
Polymorphous low‐grade neuroepithelial tumor of the young diffuse	1
Diffuse low‐grade glioma, MAPK pathway‐altered	
Pediatric‐type diffuse high‐grade gliomas diffuse	
Diffuse midline glioma, H3 K27‐altered diffuse	
Diffuse hemispheric glioma, H3 G34‐mutant diffuse	4
Diffuse pediatric‐type high‐grade glioma, H3‐wild type and IDH‐wild type	
Infant‐type hemispheric glioma circumscribed	
Circumscribed astrocytic gliomas	
Pilocytic astrocytoma	
High‐grade astrocytoma with piloid features pleomorphic	
Pleomorphic xanthoastrocytoma	2, 3
Subependymal giant cell astrocytoma	
Chordoid glioma	
Astroblastoma, MN1‐altered	

### 1.2 Standard of care

Glioblastoma current standard of care includes surgical resection of the tumor followed by radiotherapy combined with the chemotherapeutic drug temozolomide (TMZ), and further TMZ treatment after radiotherapy termination [18]. Gross‐total resection is associated with improved overall survival (OS). The initial gross‐total resection, if feasible, allows not only tumor volume reduction but also tumor genotyping and a proper histological diagnosis [19]. Magnetic resonance imaging 48 h post operation allows the establishment of a baseline program for subsequent therapeutic treatments.

Radiotherapy with high‐energy beams such as X‐rays or protons, and concomitant TMZ has long been used since a phase III trial was published in 2005 [20]. TMZ (sold under the brand names Temodar, Temodal, Temcad, among others) is an alkylating agent that adds methyl groups to the DNA thus promoting DNA damage. The benefit of TMZ is mainly limited to the patients harboring gene silencing by promoter methylation of the enzyme *O*‐6‐methylguanine‐DNA methyltransferase (MGMT). MGMT silencing prevents the removal of the TMZ‐induced guanine‐alkyl groups in the DNA favoring the accumulation of DNA damage and cell death [21]. A non‐invasive anticancer therapeutic approach approved for GBM treatment in the last years is the Tumor‐Treating Fields (TTF) therapy. This therapy, approved for recurrent (in 2011) and newly diagnosed GBM (in 2015) in adults, delivers transcutaneous alternating electric fields that exert physical forces on cancer cells affecting cancer cell viability and tumor progression [22]. The seminal EF‐14 clinical trial (NCT00916409) showed significant OS in newly diagnosed GBM patients when TTF therapy was added to maintenance TMZ, with no significant increase in rates of systemic adverse events [23]. The real impact of TTF implementation on patient survival will be seen in the next years.

Despite treatment, tumor recurrence occurs within 6 months in most patients (90%) and there is not a standard of care treatment for recurrent GBM [24]. Among options, further surgical resection or chemotherapy are available. Alkylating chemotherapy with other compounds such as lomustine, carmustine or TMZ rechallenge are the options available for patients with MGMT promoter methylation [25]. The use of bevacizumab (Avastin), a drug now being used regularly as an anti‐neovascularization agent for recurrent GBM, is currently an approved treatment in the United States but not in Europe. Bevacizumab therapy for recurrent GBM has shown a moderate increase in the progression‐free survival (2.7 months) of patients after the treatment, although it has not shown OS benefit [26].

## 2 Molecular drivers of GBM

**Fig. 1:**
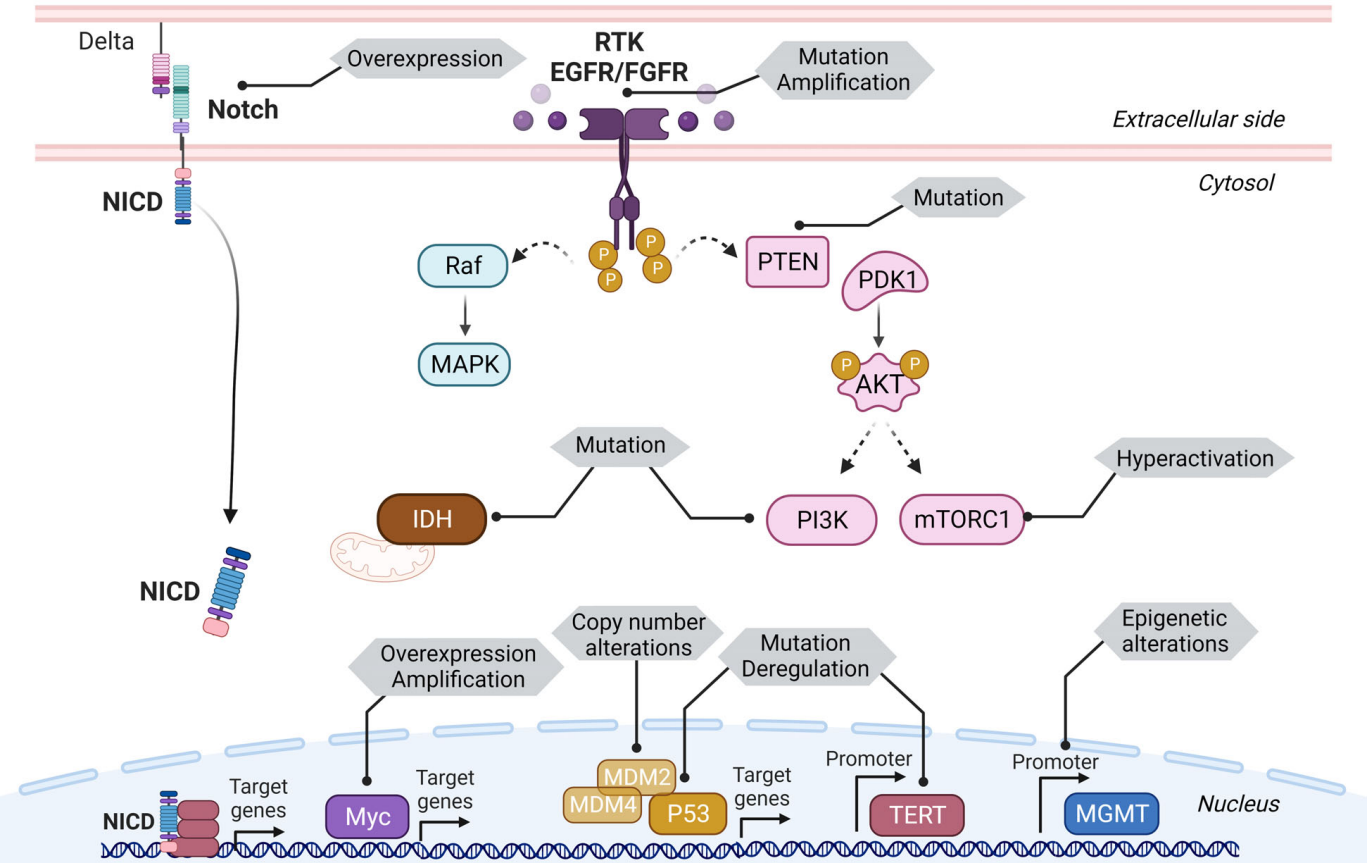
Main molecular drivers and related signaling pathways affected in GBM highlighting the principal alterations associated. Mutations in the *EGFR*, *PTEN*, and *TP53* genes are the most common mutations in primary GBM. *IDH* mutations occur predominately in secondary GBM and are associated with improved survival. Other common molecular alterations consist of copy number modifications and epigenetic changes such as gene loss for the p53‐regulator *MDM2* or the methylation of the *MGMT* promoter, respectively. Several factors within signaling pathways are overexpressed or highly activated in GBM including Myc, mTORC1 and Notch signaling. GBM, glioblastoma. Created with Biorender.com.

Cancer driver genes are considered to be genes whose acquired mutations provide a cell growth advantage resulting in tumorigenesis or cancer progression. Mutations in the *EGFR*, *PTEN*, and *TP53* genes are the most common mutations in primary GBM [27] (Fig. 1). The cell of origin of GBM is still controversial with evidence supporting either a glial cell or a neural stem cell (NSC) origin [28]. Recent analyses using laser microdissection and deep sequencing of triple‐matched tissues pointed at NSCs from the subventricular zone (SVZ) as the cells of origin [29]. This study has shown that NSCs from the tumor‐free SVZ of GBM patients harbor somatic mutations also found in the matched tumor. Despite an average of 15.6 somatic mutations were shared between the tumor‐free SVZ and the matched tumor per individual, the tumor‐free SVZ tissue contained low level of driver mutations within the known GBM‐driving genes (EGFR, PTEN, TP53). Single‐cell cloning and targeted sequencing suggested the cells containing driver mutations in tumor‐free SVZ tissue would clonally evolve into GBM [29]. Interestingly, these shared somatic mutations were only found in patients with wild‐type *IDH*.

Mutations in the *IDH* gene are used to distinguish between primary (*de novo*) and secondary GBM, as described in the 2021 WHO Classification of Tumors of the Central Nervous System [12]. *IDH* mutations account for 73% of secondary GBM whereas in primary GBM these mutations are found in less than 4% of the clinical cases [30]. Therefore, the majority of primary GBM patients have *IDH*‐wild type while *IDH* mutations are associated with improved survival. IDH catalyzes the oxidative carboxylation of isocitrate to α‐ketoglutarate participating in glutamine metabolism, the tricarboxylic acid cycle (TCA), lipogenesis and redox homeostasis [31]. *IDH* mutations disrupt the conversion of isocitrate to α‐ketoglutarate, favoring the production of d‐2‐hydroxyglutarate, an oncometabolite that promotes oncogenesis [32]. It results in a metabolic reprogramming in *IDH*‐mutated glioma. In addition, IDH‐mutant GBM presents a glioma‐CpG island methylator phenotype. Through DNA hypermethylation, the expression of several genes is epigenetically altered in these gliomas [33]. IDH1 promotes GBM cell growth *in vitro*, and the suppression of its activity decreases lipid biosynthesis, alters redox homeostasis by increasing ROS production, and promotes cellular differentiation. All of the above contribute to an impaired tumor growth in mouse models [34].

The well‐known p53 tumor suppressor is a sequence‐specific nuclear transcription factor that is activated in response to cellular stress to facilitate DNA repair or to induce cell death. *TP53* is one of the most common deregulated genes in cancer. Indeed, the p53 pathway is deregulated in 85% of GBM according to The Cancer Genome Atlas [34]. The main regulators of p53 expression and function are MDM2 and MDM4 proteins. Both proteins are p53 negative regulators that can work together and independently to inhibit and degrade p53. One of p53 main effector genes is *p21*, which blocks cell cycle progression by inhibiting the function of cyclin‐D proteins [35].

Additional somatic mutations have been found in the *TERT* gene promoter, *NF1* and in the catalytic and regulatory subunits of the PI3K heterodimer genes (*PIK3CA*, *PIK3R1*). Other molecular alterations such as copy number modifications (*MDM2* loss, Chromosome 7 gain, Chromosome 10 loss), gene overexpression or amplification (EGFR, PDGFRA, Notch, c‐Myc, FGFR) and epigenetic changes such as methylation (*MGMT* promoter methylation), are also involved in the pathogenesis of GBM [36].

Alterations in signaling pathways associated to the receptor tyrosine kinase superfamily (RTK) are present in 88% of GBM. Amplification of the RTK transmembrane glycoprotein EGFR is present in more than 60% of GBM, and approximately 50% of them harbor a unique mutant variant, EGFRvIII [37]. EGFRvIII is a constitutively active version of the receptor due to the lack of the extracellular ligand‐binding domain. EGFR amplification is associated with GBM progression, promoting invasion, proliferation and resistance to treatment [38]. Mutations and alterations either in the tumor‐suppressor PTEN or in upstream components of the RTK pathway, result in a constitutive activation of the PI3K pathway. PI3K, which belongs to a family of lipid kinases, is involved in growth, survival, and cell metabolism. Indeed, GBM shows an increase in PI3K/AKT‐downstream signaling pathways, including the mammalian target of rapamycin (mTOR) and the Myc oncogenic transcription factor pathways [39]. A small subset of primary GBMs (3–8%) harbors chromosomal translocations that generate a fusion protein containing FGFR and transforming acidic coiled‐coil (TACC) protein domains (FGFR‐TACC), with oncogenic transforming properties and constitutively active RTK [40, 41].

Among the not mutated but deregulated signaling pathways, a plethora of studies confers an important role to Notch pathway in GBM development. The Notch signaling pathway has been extensively studied both in GSC biology and in GBM oncogenesis and progression due to its role in tissue development and stemness regulation [42, 43]. Indeed, Notch signaling has been implicated in mechanisms of radio‐ and chemoresistance of GBM in both cellular and *in vivo* models [44, 45]. Several lines of evidence also suggest that Notch deregulation confers advantages for GBM initiation by favoring stem cell features and apoptosis resistance mechanisms in NSC [46].

## 3 Metabolic adaptation in GBM

Tumor metabolism reprogramming is one of the hallmarks of cancer [47]. Tumor cells have to adapt to the high demand for energy, lipids, proteins, sugars, and other molecules to meet cell proliferation, growth, and motility requirements. Tumor growth relies mainly on both glucose and glutamine metabolism for energy production and anabolism with certain particularities related to the tissue of origin [48]. Metabolism, signaling, and cellular activities are finely coordinated and mutually regulated to sustain tumor homeostasis.

**Fig. 2:**
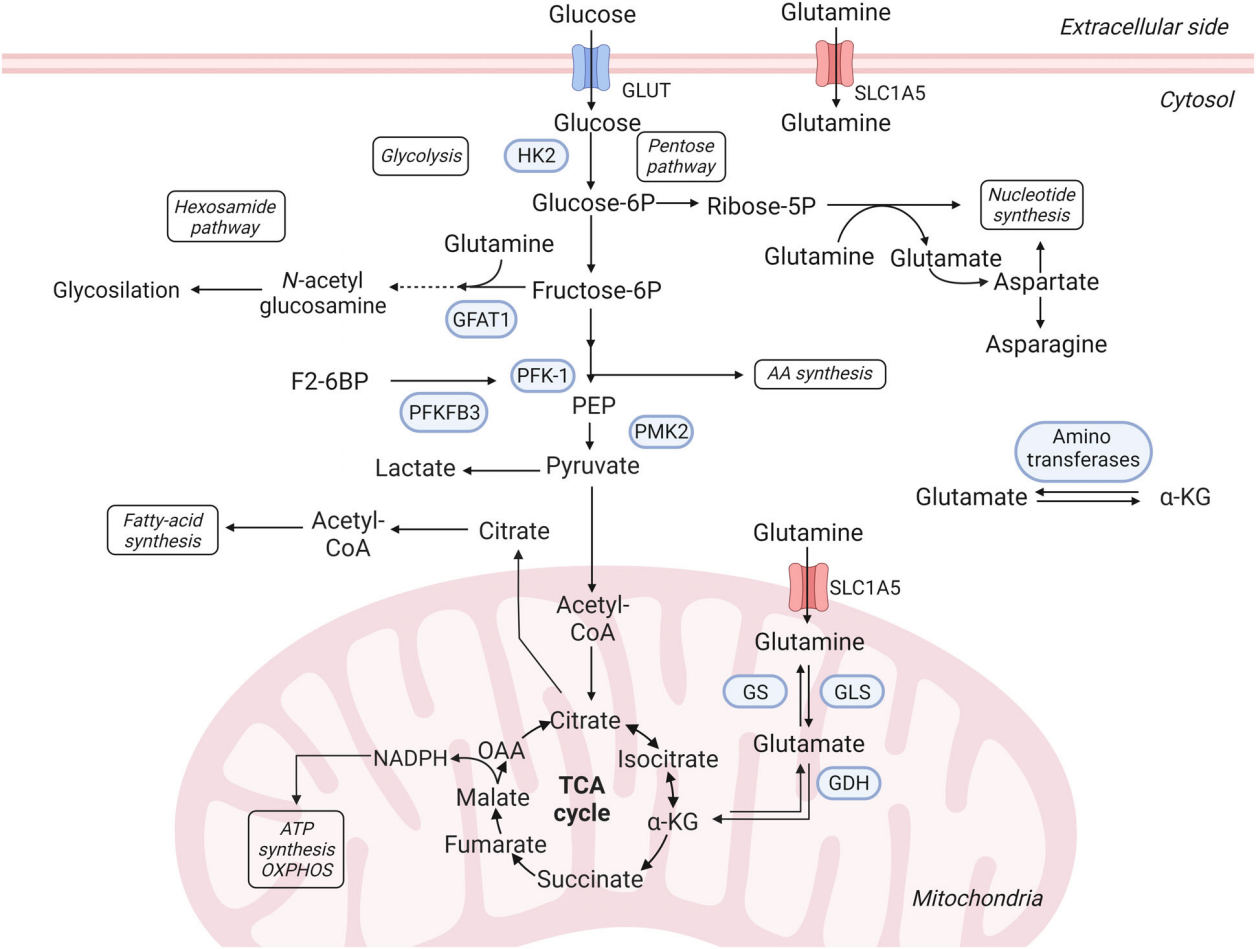
Glutamine and glucose metabolism. Glucose is the main energy source in the brain. Glycolysis transforms glucose to pyruvate followed by acetyl‐CoA production to feed TCA cycle for ATP synthesis through mitochondrial respiration. GBM cells divert most of the pyruvate to aerobic glycolysis for ATP synthesis and the end‐product lactate is secreted. Glutamine supplies carbon and nitrogen to fuel biosynthesis through several pathways including hexosamide pathway and pentose pathway. Glutamine also enters the mitochondria to supply the TCA cycle intermediate α‐KG through glutaminolysis for ATP production. GBM generally exhibit metabolic reliance on glutamine but limited glutamine availability enhances a metabolic reprogramming to sustain GBM growth favoring glutaminogenesis. TCA, tricarboxylic acid cycle. Created with Biorender.com.

**Fig. 3:**
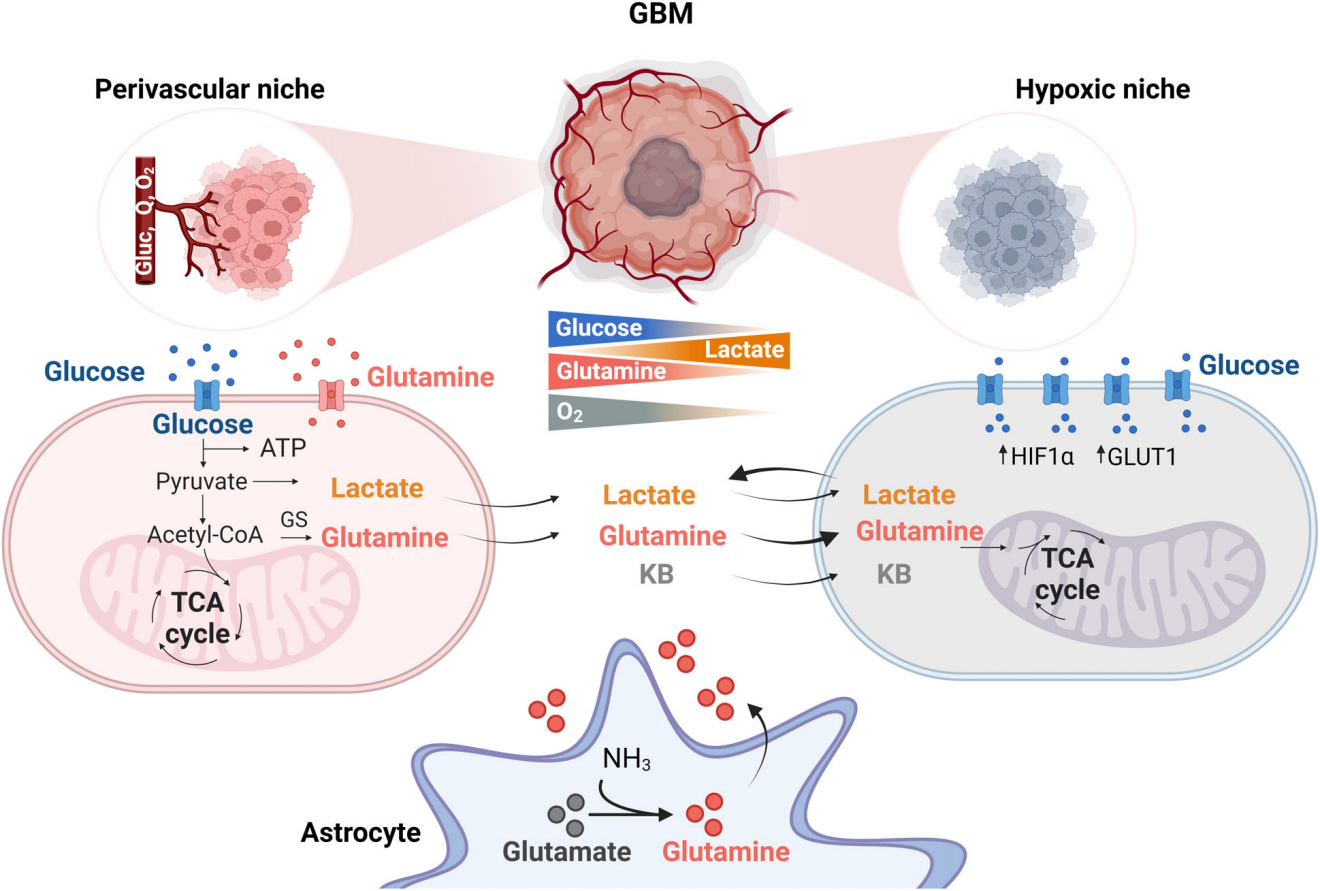
Metabolic adaptation to changes in nutrient and oxygen availability within GBM. Perivascular niche displays an enhanced glycolytic pathway to TCA cycle based on blood glucose availability. In contrast, cells from hypoxic areas employ glutamine, lactate and potentially ketone bodies (KB) from the microenvironment as energy substrates to supply a truncated TCA cycle. Hypoxia also enhances anaerobic glycolysis optimizing glucose uptake and realizing lactate to be used as carbon source by neighbor tumor cells. Astrocytes provide glutamine and KB to the tumor microenvironment. TCA, tricarboxylic acid cycle. Created with Biorender.com.

The brain's main energy source is glucose, consuming approximately 20% of the body's daily glucose utilization [49]. Glucose can either be metabolized or partially stored as glycogen primarily in astrocytes. Glycolysis converts glucose into pyruvate and then pyruvate is used either for oxidative phosphorylation (OXPHOS) at the mitochondria or fermented into lactate in the cytosol (Fig. 2). Tumor cells prefer to transform glucose to lactate as the main energy source even in aerobic conditions since it is the most time‐effective method of glucose consumption, despite a lower yield of ATP production per reaction compared to OXPHOS. This alteration known as the Warburg Effect or aerobic glycolysis, is one of the best described metabolic adaptation in tumor cells. Indeed, aerobic glycolysis is three times higher in GBM than in normal brain tissue [50]. However, despite the importance of glycolysis levels, GBM cells also utilize mitochondrial glucose oxidation during aggressive tumor growth *in vivo* [51]. In order to supply the high demand of glucose into the cancer cells, the upregulation of glucose transporters (GLUT1‐4) has been described in several cancer types including GBM [52]. GLUT1 and GLUT3 expression seems to be particularly related to oxygen levels and therapy resistance in GBM. The hypoxic environment in the intermediate and core areas of the GBM leads to the stabilization of the hypoxia inducible factor‐1α (HIF‐1α), the transcription factor controlling the expression of genes involved in the hypoxic response. Upon stabilization, HIF‐1α increases GLUT1 expression (Fig. 3), to increase glucose uptake, as well as hexokinase 2 (HK2), pyruvate kinase and lactate dehydrogenase A (LDHA) to maintain glycolysis [53]. Under hypoxic conditions, HIF‐1α also activates p21 by a direct transcriptional regulation, and inversely p21 promotes HIF‐1α expression, showing a reciprocal positive feedback loop that enhances radioresistance in hypoxic conditions in cellular and in xenograft GBM models [54]. In the proposed mechanism, HIF‐1α/p21 communication results in the upregulated expression of glycolytic enzymes such as GLUT1 or LDHA that favors a metabolic state necessary for radioresistance. Indeed, GLUT1 or LDHA knockdown sensitizes GBM cell lines to radiation under hypoxic conditions, reducing viability and clonogenicity capacity [54].

Although the brain uses glucose as the major energy substrate, it can also transiently use other sources, including ketone bodies, glutamine, fatty acids or acetate. In the healthy brain, glutamine participates in the glutamine‐glutamate cycle [55]. This cycle is coordinated between neurons and astrocytes so the glutamate released by neurons is incorporated by astrocytes to produce glutamine by the glutamine synthetase (GS). Astrocytic glutamine is then released and taken up by neurons to produce glutamate. In GBM, there are glutaminogenic tumor cells that are crucial to sustain glutamine requirements in nutritional restricted regions of the tumor (Fig. 3). Surrounding astrocytes can also contribute to the tumor's needs of glutamine by the synthesis and secretion of glutamine [56]. Glutamine metabolism fuels the biosynthetic machinery of the tumor cells for the synthesis of nucleotides [56], fatty acids [57], and amino acids [58], fundamental building blocks for tumor cell proliferation and growth (Fig. 2). Glutamine also feeds the TCA cycle providing α‐ketoglutarate (αKG) by two deamination reactions named glutaminolysis, performed by the glutaminase (GLS) followed by the glutamate dehydrogenase (GDH). This anaplerotic role of glutamine contributes to the TCA cycle production of the redox cofactors FADH_2_ and NADH. Glutamine‐derived glutamate and αKG are also used to generate other amino acids through transamination (serine, alanine, aspartate, and asparagine), for nucleotide synthesis, oxidative stress control, and mTOR activity for cell growth. Both glutaminolysis and transamination are favored by the overexpression of c‐Myc, while K‐RAS oncogene activity favors glutaminolysis rather than transamination [59]. GS activity also links glutamine metabolism to radiotherapy response. Radiation promotes metabolic reprogramming with an increase in GS expression that enhances glutamine production. This induction of glutaminogenesis in irradiated cancer cells serves to fuel cellular nucleotide synthesis for a more efficient DNA repair to facilitate growth under radiation stress. Indeed, shRNA‐mediated loss‐of‐function of GS in U251 cells leads to radiosensitization by increasing irradiation‐induced apoptosis [60].

The contribution of ketone bodies through fatty acid metabolism to sustain energy production for GBM adaptation to glucose limitation is still not clear. Some early studies describe the inability of GBM cells to compensate for glucose restriction by metabolizing ketone bodies in cellular models, as well as an unrestricted ketogenic diet in GBM xenografted mice show no beneficial effect on animal survival [61]. On the contrary, more recent results indicate a capability of GBM to use the ketone bodies to sustain growth by showing that both cell cultures and orthotopic xenografted tumors are able to proliferate and grow under ketone supplementation and unrestricted ketogenic diet respectively under low glucose availability [62]. Discrepancies between studies may reside in the cellular and animal models used and in the absence of a consensus on the ketogenic diet or concomitant cancer therapy applied. Indeed, using the ketogenic diet for the treatment of patients with GBM is still exploratory due to the absence of a clear survival benefit shown by any clinical trials' intervention plan and the inconsistency in patient response.

Probably due to its astroglial lineage, GBM can also completely oxidize acetate as astrocytes do to support neuronal function under limited glucose supply [63]. Mice harboring human GBM or brain metastases oxidize acetate in the tumors as a result of the nucleo‐cytosolic acetyl‐CoA synthetase enzyme (ACSS2) upregulation [64]. Indeed, the expression of ACSS2 is directly related with GBM growth and its malignant potential since ACSS2 inhibition by shRNA is able to induce cell death in primary GBM spheres [64].

Recent studies show that GBM cells are able to adapt to glucose deprivation using lactate. Lactate is able to sustain cell survival and promote cell invasion of GBM tumorspheres and orthotopic GBM xenografts by enhancing oxidative metabolism in a glucose‐deprived context [65]. The extracellular lactate is taken up by the cells and converted to pyruvate to fuel the TCA cycle and also to produce acetyl‐CoA for histone acetylation purposes (Fig. 3). A model of metabolic symbiosis between GBM cell populations has been proposed based on the preferential activity of LDH to either produce (LDHA) or a consume lactate (LDHB) [65].

Differential intratumoral metabolism has also been observed between GSC and the bulk tumor cells. GSCs appear to be less glycolytic with a higher mitochondrial reserve capacity than differentiated glioma cells, which positively correlates with a resistance of the GSC population to ionizing radiation [66]. Microenvironment differences can also modulate GSC metabolism within the tumor. GSCs located in more hypoxic and nutrient restricted areas increase the use of glutamine as energy substrates while perivascular GSCs maintain an enhanced glycolysis [48, 67] (Fig. 3).

Metabolic phenotype has been correlated with GBM molecular subtypes in terms of glutamine low or high consumption. The proneural subtype and the more aggressive mesenchymal subtype coincides with the so‐called glutamine‐low and the glutamine‐high respectively, reaffirming the hypothesis that aggressiveness/invasion is linked to metabolic adaptation [67]. Indeed, glutamine‐low are GS‐positive cells that employ glutamine mainly for nucleotide synthesis and other anabolic processes, unlike glutamine‐high cells, which are GS‐negative cells that rely on external glutamine availability for anaplerosis [56].

## 4 Communication between metabolism and signaling in GBM homeostasis and chemoresistance

Signaling pathways sensing external and internal cell status have an impact on the metabolic response and cellular functions. Similarly, metabolic state also influences cellular activity through the communication with signaling pathways. The response to therapy‐induced cytotoxicity requires a precise crosstalk between signaling and metabolic activity to provide the right response to eliminate cytotoxic stress and restore functional and metabolic homeostasis. This section recapitulates data supporting the communication between signaling and metabolism to support tumor progression and treatment resistance in GBM. Signaling modifications in pathways such as Ras/MEK, PI3K/AKT, Notch, mTOR, c‐Myc, NFκB, HIF1α, as well as in epigenetic regulatory mechanisms, contribute to the reprogramming of the metabolism of certain nutrients and therefore to the plasticity and metabolic adaption of GBM [64].

### 4.1 Glutamine and glucose metabolism

**Fig. 4:**
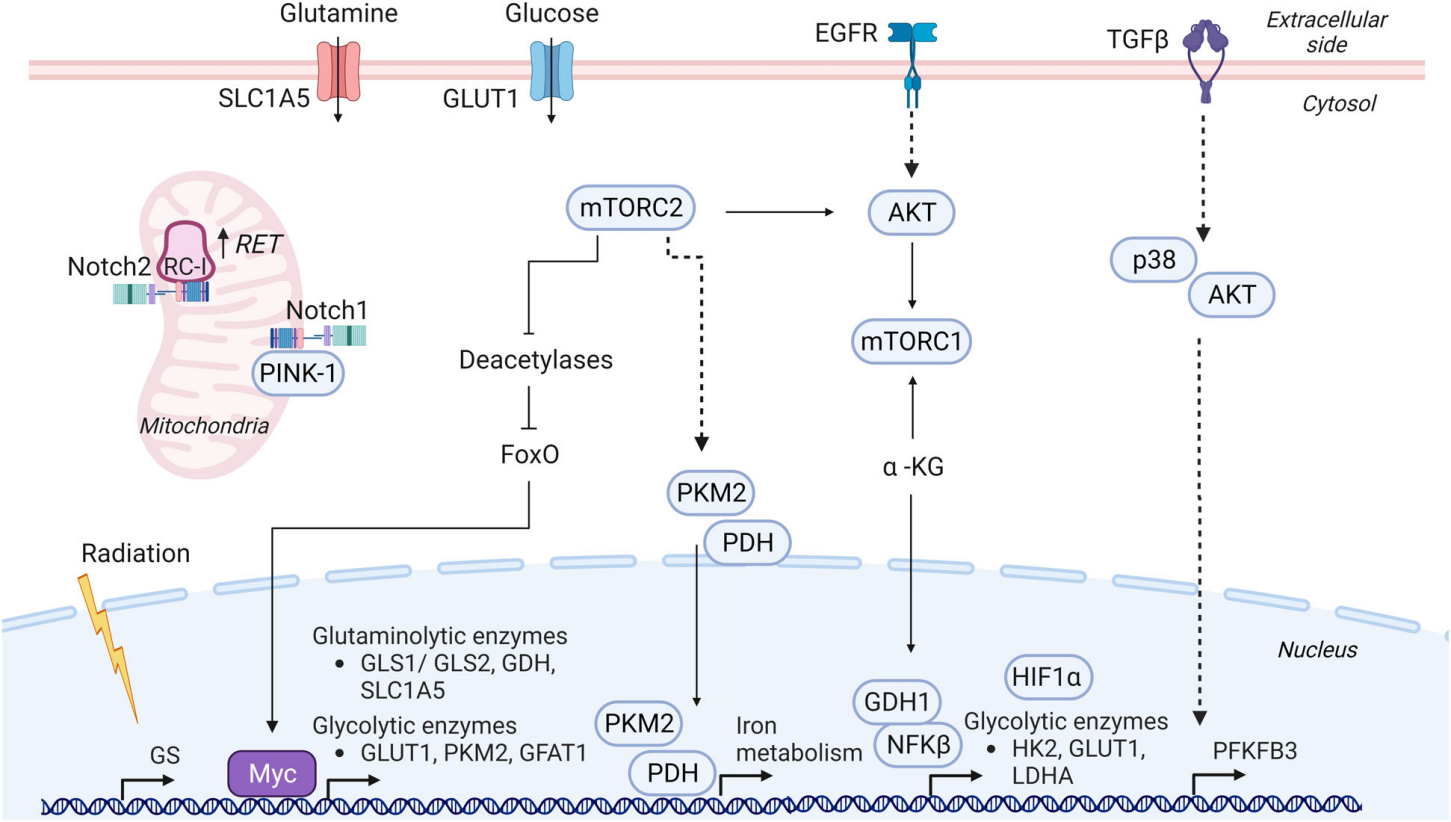
Communication of glutamine and glucose metabolism with signaling pathways in GBM. Extracellular and intracellular changes lead to interactions between metabolic and signaling elements for an intracellular reprogramming to sustain energy production and biosynthesis. Created with Biorender.com.

Glutamine‐derived αKG has been described as a signaling molecule necessary for the activation of mTORC1 kinase activity. The mTORC1 pathway regulates cell growth by sensing, among others, cell nutritional state through the levels of αKG. Upon activation, mTORC1 promotes growth through protein translation, ribosomal activity and autophagy inhibition [68, 69]. Autophagy inhibition due to an anomalous αKG‐mediated activation of mTORC1 in nutrient limitation conditions generates a cellular imbalance leading to a type of apoptosis named glutamoptosis [70]. The dependency of tumor cells on glutamine availability, provided either by neighbor cells or through the blood stream [56] can be modulated by mTOR activity. Treatment of glutaminolytic U87 GBM cell line with mTOR inhibitors, either in cell culture or after subcutaneous xenografting, induces a decrease in glycolytic enzyme expression and an increase in GLS expression and intracellular glutamate levels [66]. This response to mTOR inhibition is described as a compensatory mechanism to sustain TCA cycle through glutaminolysis when glycolysis is compromised. Accordingly, glutamine deprivation or GLS inhibition in combination with mTOR inhibitors induces apoptosis in U87 cells, an effect that is rescued by αKG addition [66]. Glutamine and glucose metabolism are also closely related by other signaling mechanisms. For example, α‐KG promotes glucose uptake through NF‐κB pathway activation. Under low glucose conditions, GDH1 interacts with NF‐κB complex components RelA and IKKβ, and the α‐KG produced by GDH1 binds and activates IKKβ to finally promote glucose uptake by GLUT1 transcriptional expression (Fig. 4) [71]. This signaling‐metabolic crosstalk results in tumor cell survival and gliomagenesis as shown in GBM cell lines and in tumor growth of orthotopic xenografts respectively.

Other GBM tumors are able to activate glutaminogenesis to sustain tumor survival by the production of their own glutamine mainly by GS activity. GS activity determines the level of dependency on external glutamine availability and the consequent glutaminolysis addiction. Paired populations of differentiated cells and GSCs generated from primary patient‐derived GBM samples show a marked difference in GS activity. The expression of GS was higher in all GSCs compared to differentiated cells and while glutamine deprivation reduced the proliferation in differentiated cells, GSCs sustained proliferation independently of glutamine supplementation [56]. Little is known about the signaling pathways involved in GS expression and activity in GBM. In T‐cell acute lymphoblastic leukemia (T‐ALL), a dependency of GS levels on Notch1 activity was demonstrated with consequences in glutamine metabolism and survival both *in vitro* and in a Notch1‐driven leukemia mouse model [72]. This study showed that high Notch1 activity induces glutamine addiction by promoting the proteosomal system activity to prevent the accumulation of GS in the absence of glutamine. Little is known about this potential Notch/GS crosstalk in GBM. However, a few studies describe a connection of Notch signaling with glutamine metabolism as well as with other elements of GBM metabolism. The pharmacological inhibition of Notch with a γ‐secretase inhibitor alters the metabolome of tumorspheres derived from both GBM cells lines and GBM cells established from primary GBM specimens. Notch blockade induces a decrease in glutamate levels as well as a general reduction of genes involved in glutamate homeostasis, including GLS [73]. Two other studies have related Notch signaling with mitochondrial activity. A study using fly genetics and patient‐derived GBM tumorspheres showed the Notch protein localized at the mitochondria outer membrane and in colocalization with the mitochondrial quality‐control kinase PINK‐1. The results from this study point to an implication of the Notch/PINK‐1 connection in mitochondrial integrity of human primary GSC cell proliferation and self‐renewal properties [74]. In support of those observations, a recent study also showed Notch2 protein localized at the mitochondria in GBM cell lines and patient‐derived GBM cells [75]. Following pharmacological and genetic approaches, this study proposed Notch2 as a regulator of the reverse electron transfer (RET) through its direct interaction with some respiratory complex I proteins (Fig. 4) [75]. Pharmacological interference of this interaction induced RET inhibition and GBM growth impairment in an *in vivo* orthotopic model. This noncanonical Notch signaling highlights the direct communication of signaling components with the metabolic machinery, bypassing a transcriptional‐mediated response for certain effects.

Myc‐driven cancer cells exhibit enhanced glutamine utilization accompanied by increased expression of glutaminolytic enzymes, including GLS1/GLS2 and GDH. Myc is implicated in glutaminolysis regulation in GBM by direct transcriptional activity. Myc inhibition by shRNA in the Myc‐amplified pediatric GBM cell line SF188, led to a significant reduction in glutamine consumption and lower levels of the high affinity glutamine importers SLC1A5 and SN2 [76]. The recycling of another glutamine transporter, the SLC38A1, by the endosomal cargo recycling system promotes TMZ resistance in GBM [77].

Myc activity also mediates an increase of the glycolytic flux through NAD^+^ metabolism in GBM cells [78]. Myc‐transduced U87 cells cultured with labeled glucose show a significant isotope enrichment in glycolytic metabolites and increased expression of several glycolytic enzymes that is dependent on a threshold of NAD^+^ levels. This Myc‐mediated glycolysis enhancement favors glucose addiction and renders GBM cells vulnerable to glycolytic inhibitors in patient‐derived GBM tumorspheres and in orthotopic GBM xenografts [78]. Myc also enhances GBM glycolysis indirectly by upregulating the transcription of the pyruvate kinase isoform PKM2‐promoting splicing factors PBT, hnRNPA1 and hnRNPA2 [79, 80]. Conversely, PKM2 nuclear kinase activity and its dephosphorylation by the cell cycle‐related CDC25A phosphatase, is necessary for β‐catenin transactivation and c‐Myc protein expression, promoting glycolysis and *in vivo* tumor growth of U87 orthotopic xenografts [81].

Myc also cooperates with mTORC2 in the crosstalk between glycolysis and glutaminolysis in GBM cells through the regulation of the glutamine‐fructose‐6‐phosphate transaminase (GFAT1). GFAT1 catalyzes the synthesis of glucosamine‐6‐phosphate and glutamate using fructose‐6‐phosphate from glycolysis and glutamine as substrates (Fig. 2). mTORC2 promotes GFAT1 activity by direct protein interaction and by promoting Myc‐mediated GFAT1 transcription [82]. A previous study in GBM cell lines also showed that cell survival depends on the communication between mTORC2 and Myc to sustain glycolysis. In this study, mTORC2‐mediated inhibition of deacetylases promotes FoxO1 and FoxO3 acetylation that leads to the release of Myc from a suppressive state (Fig. 4) [83]. Of note, mTORC2 also promotes GBM survival by an epigenetic regulation of iron metabolism [84]. By a gain/loss of function approach in GBM cellular models, mTORC2 has been implicated in histone acetylation of iron metabolism‐related genes through the promotion of nuclear translocation of two acetyl‐CoA‐producing enzymes, pyruvate dehydrogenase (PDH) and PKM2, to increase nuclear acetyl‐CoA levels for acetylation. Indeed, mTORC2 inhibition by Rictor knockdown compromises iron intracellular levels and proliferation of U87 GBM cell line.

Glycolysis is also controlled by TGF‐β1 via the upregulation of the enzyme PFKFB3. This enzyme is a critical regulator of glycolysis since its product, fructose 2,6‐bisphosphate, is the most potent allosteric activator of the glycolytic rate‐limiting enzyme PFK1 [85]. Treatment of GBM cells T98 and U87 with TGF‐β1 upregulates PFKFB3 mRNA and protein expression resulting in an increase in fructose 2,6‐bisphosphate concentration, glucose uptake, glycolytic flux and lactate production. Following a pharmacological approach, this study also describes a communication of TGF‐β1 with p38 MAPK and PI3K/AKT signaling pathways for PFKFB3 upregulation (Fig. 4) [86].

Glioblastoma tumors also consume acetate to generate acetyl‐CoA through ACSS2 enzymatic activity for energetic and lipid production purposes. A recent study has established a communication between ACSS2 and kinase activity that regulates ACSS2 activity in GBM growth. The enzyme *O*‐GlcNAc transferase (OGT), catalyzes intracellular glycosylation of serine and threonine residues with the addition of *N*‐acetylglucosamine as posttranslational modifications of nuclear and cytoplasmic proteins. This study shows that OGT activity is necessary for the CDK5 kinase‐mediated phosphorylation of ACSS2 that promotes its stability and reduced degradation (Fig. 4). Inhibition of OGT or CDK5, or blocking ACSS2 phosphorylation site, impair GBM growth in *in vivo* orthotopic xenografts [87].

### 4.2 Lipid metabolism

Lipids are essential components of the brain, which account for about 50% of its weight. Lipids, including fatty acids, triglycerides, phospholipids, and cholesterol, are soluble organic compounds with essential structural, signaling, and energetic functions. Due to the high rate of proliferation, GBM cells depend on lipid biosynthesis for the high demand of these components in the membrane and in signaling pathways [88].

**Fig. 5:**
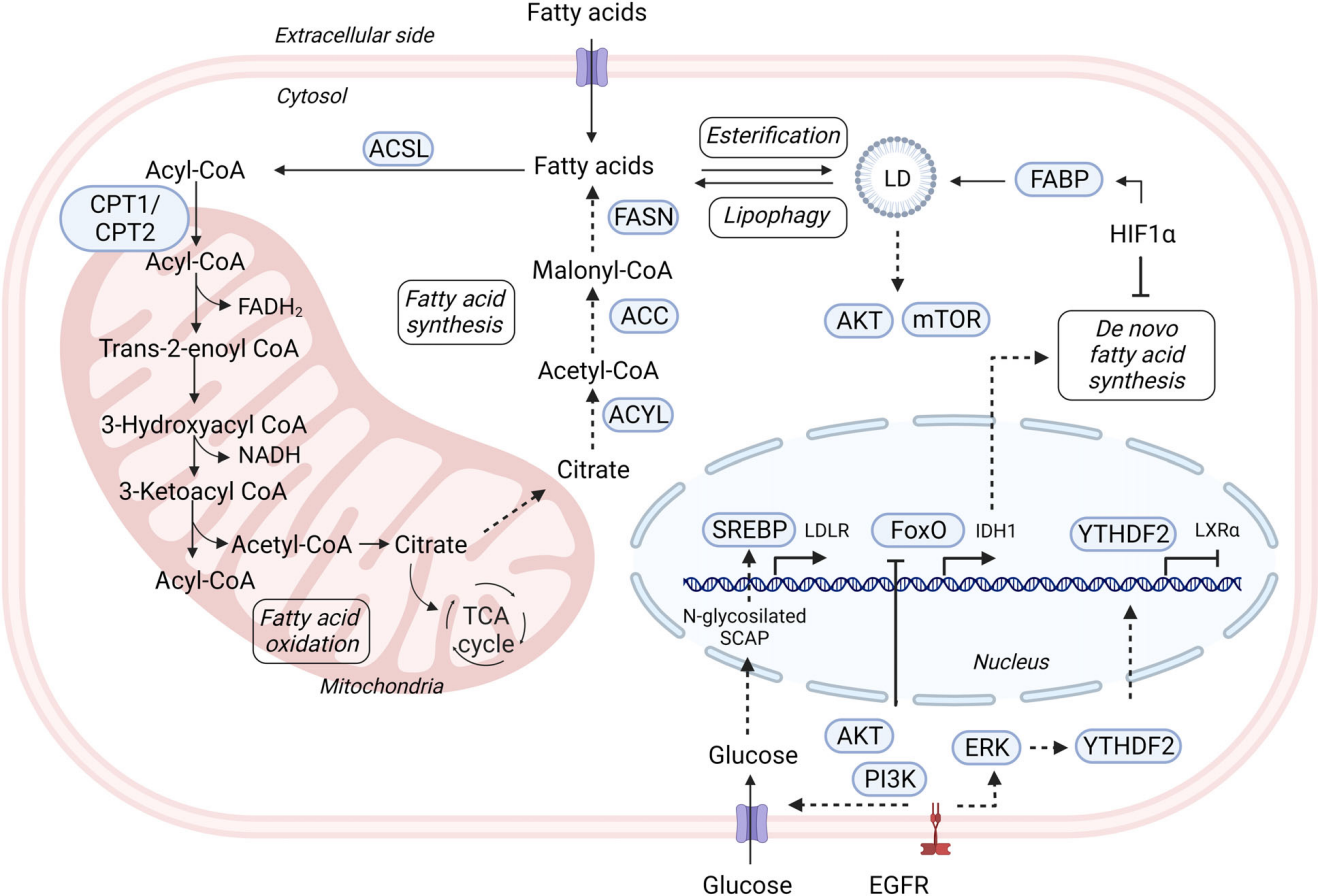
Interaction between lipid metabolism and signaling pathways in GBM. Both growth factor‐ and hypoxia‐related signaling pathways interact with the intracellular processes involved in fatty acid and cholesterol synthesis and utilization at the gene expression and protein activation/modification levels. GBM, glioblastoma. Created with Biorender.com.

An enhanced capacity for *de novo* lipid synthesis is a metabolic feature of most cancer cells, including GBM. The first step of the *de novo* fatty acid synthesis is the production of cytosolic acetyl‐CoA, via ATP citrate lyase or from ACSS2. This is followed by the ATP‐dependent carboxylation of the cytosolic acetyl‐CoA by acetyl‐CoA carboxylase (ACC), generating malonyl‐CoA and palmitate in a reaction catalyzed by fatty acid synthase (FASN) (Fig. 5). The *de novo* lipogenesis is implicated in the metabolic adaptation to promote GBM proliferation and growth. Indeed, the genetic inhibition of ACC or the pharmacological and genetic inhibition of FASN induces proliferation arrest in GBM patient‐derived GSC lines [89]. A relevant communication between lipid metabolism and signaling has been described in GBM involving EGFR, glucose and the transcriptional regulator of *de novo* lipogenesis so‐called sterol regulatory element‐binding protein‐1 (SREBP1). In the described mechanism using the U87 GBM cell line, EGFR promotes SREBP1 activation indirectly through the stimulation of glucose import, necessary for the N‐glycosylation of the SREBP1 activator SCAP. The impairment of SCAP N‐glycosylation in GBM cells with activated EGFR signaling inhibits SREBP1 activation, reduces tumor growth of orthotopic GBM xenografts, and extends mice survival (Fig. 5) [90].

Fatty acid oxidation (FAO) is also adapted in GBM. FAO also known as fatty acid β‐oxidation, is a multistep process that occurs at the mitochondria, where fatty acids are oxidized and broken down into their carbon substrates [91] (Fig. 5). The redox power generated as FADH2 and NADH by FAO and by acetyl‐CoA oxidation in the TCA cycle, is employed for ATP synthesis during OXPHOS, but also in other processes such as amino acid or nucleotide synthesis [91]. Acyl‐CoA‐binding protein (ACBP) promotes FAO in GBM by controlling the availability of acetyl‐CoA to mitochondria. Besides, some *in vivo* studies show that the knockdown of carnitine palmitoyltransferase 1A (CPT1A), a rate‐limiting enzyme allowing the transport of acyl‐CoA to mitochondria, reduces GBM growth and increases survival [62].

Fatty acid oxidation is involved in TMZ resistance of GBM through the activation of the Sp1 transcription factor [92]. Sp1 increases the expression of prostaglandin‐endoperoxide synthase 2, that leads to PGE2 (prostaglandin E2) production in recurrent GBM patients and in TMZ‐resistant GBM cells. The Sp1‐mediated production of PGE2 after TMZ treatment enhances mitochondrial FAO and TCA cycle by increasing mitochondrial fusion. In this context, it is relevant to mention that Sp‐1 gene expression is regulated by cell cycle regulatory proteins binding to Sp‐1 promoter, such as p21 and p53 [93]. Therefore, TMZ insult on DNA replication and cell cycle has an impact on Sp‐1 expression that will promote lipid metabolism in a PGE2‐dependent manner to overcome TMZ cytotoxicity.

Isocitrate dehydrogenase‐wild type, frequently overexpressed in GBM, participates in the metabolic adaptation of GBM to support its aggressive growth and therapy resistance. Indeed, the inhibition of IDH1, both genetic or pharmacologically, leads to a decrease in GBM cell growth and higher sensitivity to RTK‐targeted therapies, increasing the survival of mice with patient‐derived GBM cells xenografts [34]. In the proposed mechanism, lipid metabolism is affected by IDH1 because NADPH levels produced during IDH1 enzymatic activity are decreased upon IDH1 suppression, limiting therefore NADPH availability for fatty acid synthesis. This study also showed that the IDH1‐mediated increase in fatty acid synthesis is a mechanism of resistance to RTK inhibitors in GBM. Comparing EGFR‐amplified and non‐amplified GSCs, the study revealed that the RTK inhibitors lead to the blockade of the AKT‐mediated inhibitory phosphorylation of FoxO, therefore promoting FoxO‐induced expression of IDH1 and consequently favoring lipid metabolism for adaptation (Fig. 5). In another study, the knockdown of wild‐type IDH in GBM cell lines and in GBM xenograft models also reduced NADPH levels and NADPH‐dependent metabolites such as deoxynucleotides and glutathione, correlating with an improvement in the therapeutic response to radiotherapy [76].

The intrinsic presence of lipid droplets (LD) has been widely reported to be a characteristic of chemoresistant cancer cell lines. LD content contributes to radioresistance and chemoresistance in breast and myeloid leukemia cancer cells [94]. LD not only sustain energy demands and biomass providing fatty acids and controlling cholesterol levels, but also regulate several prosurvival signaling pathways. Indeed, the increase in LD in myeloid leukemia U937 cells after recurrent treatment with an aminopeptidase inhibitor is accompanied by an activation of AKT/mTOR pathway, highlighting the communication between metabolism and signaling in therapy resistance [95]. In GBM, triglycerides, the major components of LD, serve as a critical energy reservoir to support GBM cell survival. When glucose level decreases, ld‐triglycerides levels also decrease and fatty acids are mobilized into the mitochondria for β‐oxidation and energy production [96]. A xenograft model of antiangiogenic therapy for GBM proposes an oxygen‐dependent mechanism of metabolic adaptation through LD accumulation after bevacizumab treatment [97]. HIF‐1α upregulation in hypoxic areas of 3D spheroids of U87 cells, promotes the accumulation of LD by inducing the activation of FABP3/FABP7‐dependent fatty acid uptake while *de novo* fatty acid synthesis is repressed in hypoxia (Fig. 5). Prevention of LD formation (using lipoprotein deficient serum) or genetic inhibition of FABP3/FABP7 to prevent fatty acid uptake, increases ROS levels in U87 cells. In accordance with a role of LD formation in tumor development, FABP3 or FABP7‐knockout U87 cells subcutaneously xenografted show a delay in tumor growth initiation, decreased proliferation and decreased lipid staining. These observations suggest that the hypoxia‐induced LD storage provides GBM cells with a better response to ROS production in hypoxic areas and favors proliferation *in vitro* and *in vivo*, although the lack of a proper tumor reduction suggests a non‐essential role of LD storage in tumor growth *in vivo* [97].

Cholesterol homeostasis is a determinant of tumor proliferation. Alterations in the expression of genes related with cholesterol biosynthesis, as shown by RNA‐sequencing analysis, favor the survival of GBM‐bearing mice treated with dopamine receptor antagonists and radiation [98]. Moreover, cholesterol dysregulation controls invasive growth in GBM. EGFR signaling seems to have a central role in the interaction between cholesterol metabolism and signaling pathways regulating cholesterol uptake and efflux. EGFRvIII promotes the expression of the low‐density lipoprotein receptor (LDLR) through a PI3K/AKT/SREBP‐1‐dependent pathway both *in vitro* in U87 GBM cells and *in vivo* using subcutaneous xenografts from human primary GBM cells [99]. The clinical relevance of this connection is further supported by the positive correlation between the immunohistochemical stainings of elements of this signaling axis (EGFR/PI3K/AKT/SREBP‐1) and LDLR in human GBM samples. In another study, the interaction of cholesterol metabolism and signaling pathways occurs through YTHDF2, an m^6^A‐eraser, which is overexpressed in GBM and has a negative impact on patient survival [100]. This study, using patient‐derived GBM cells and GBM cell lines, demonstrates that EGFR‐mediated activation of ERK signaling is necessary for YTHDF2 stabilization, which leads to m6A‐dependent mRNA decay of the nuclear receptor LXRα resulting in reduced intracellular cholesterol availability.

Glioblastoma stem cells also show differences compared to the differentiated glioma cells in terms of lipid metabolism, particularly regarding *de novo* lipogenesis. The incorporation of glucose and acetate into lipids is higher in GSCs and seems to contribute also to GSC stemness [89]. Inhibition of fatty acid synthesis by pharmacologically targeting FASN in GSCs significantly reduces stemness markers such as SOX2, nestin or CD133 [89]. Targeting FAO via Acyl‐CoA binding protein also hinders GBM invasion of patient‐derived GBM tumorspheres and xenografts [101]. Lipid content and lipid metabolism can also differ within the tumor. Targeted lipidomic analysis revealed a shift in lipid metabolism in comparing GSCs and non‐GSCs in patient‐derived models. The high expression of fatty acid desaturase FADS1/2, which is essential to maintain GSC viability, is responsible for a decreased level of neutral lipids in GSCs [102]. A pharmacological drug targeting sterol CoA desaturase (SCD) and thus, *de novo* lipid synthesis, triggers lipotoxicity in patient‐derived GSCs, partially protected by the activation of the energy sensor AMP‐activated protein (AMPK). This study further shows that an altered MEK/ERK signaling and particularly its mediated repression of AMPK favor the vulnerability to SCD inhibitors shown by patient‐derived both GSC cultures and luciferase‐expressing orthotopic tumors [103].

### 4.3 Nucleic acid metabolism

Nucleic acid metabolism plays also a key role in GBM and its chemoresistance although less is known about the regulatory mechanisms connecting this metabolism with signaling pathways. Purine and pyrimidine biosynthetic pathways are increased in tumors to fulfill the high demands of tumor cells since both purines (nucleotides with adenine or guanine) and pyrimidines (with cytosine, thymine or uracil) are essential for the synthesis of DNA and RNA [104]. They are synthetized by *de novo* pathway or by the salvage pathway, the latter one recycling already‐present nucleotides in the cell or in the environment [104].

Indeed, the expression of the enzymes dihydroorotate dehydrogenase (DHODH) and 1‐orotate phosphoribosyl transferase and 2‐orotidine‐5′‐decarboxylase/uridine monophosphate synthetase, implicated in *de novo* biosynthesis of pyrimidines, is upregulated in GBM. Higher expression of pyrimidine synthesis genes correlates with a worse prognosis of GBM patients [105]. The inhibition of DHODH decreases ribosomal DNA transcription and leads to nucleolar stress in GBM cells and in GBM tumor xenografts. DHODH inhibition induces a decrease in proliferation of GBM cells independently of the level of resistance to TMZ, and a reduction of tumor growth *in vivo*. Of note, the inhibition of DHODH does not affect pyrimidine levels in normal brain cells and tissue, suggesting a dependency of GBM cells on the *de novo* biosynthesis but not of normal cells [106]. GSCs are also dependent on this biosynthetic pathway since targeting DHODH inhibits survival, self‐renewal and *in vivo* GBM tumor initiation [105]. The IDH‐mutant gliomas are particularly dependent on the *de novo* pyrimidine biosynthesis, since inhibitors targeting enzymes of this metabolism including DHODH, preferentially kill IDH1‐mutant glioma cells and patient‐derived IDH‐mutant GSC compared to IDH‐wild type cells. Indeed, mutant IDH activity sensitizes glioma cells to DHODH inhibition by increasing their susceptibility to replication‐dependent DNA damage caused by nucleotide pool imbalances. The model has been validated in isogenic engineered glial cells, patient‐derived tumorspheres and orthotopic xenografts [107]. DHODH activity seems to be linked to EGFR signaling in GSCs since GSCs lacking EGFR amplification or EGFRvIII mutation are more sensitive to DHODH inhibition leading to cell death, although the mechanism behind this has still not been described [108].

On the other hand, purine metabolism is also related with therapy resistance in GBM. The inhibition of guanosine triphosphate (GTP) synthesis radiosensitizes both GBM cells and patient‐derived tumorspheres by altering DNA repair mechanisms. The FDA‐approved prodrug mycophenolate mofetil (MMF) which inhibits GTP synthesis, augments the effects of radiation in a GBM orthotopic patient‐derived xenograft model, highlighting a role for purine‐metabolism in radiation resistance in GBM [109]. MMF inhibition of the inosine‐5′‐monophosphate dehydrogenase 2, a rate‐limiting enzyme in purine biosynthesis, also increases the efficacy of TMZ *in vivo*, improving the median survival of mice orthotopically xenografted with patient‐derived GBM cells [110]. Metabolomic and genomic analyses reveal specific upregulation of the *de novo* purine synthesis in GSCs from GBM patient‐derived xenografts. In this study, the communication between PI3K/c‐Myc signaling with the *novo* purine synthesis was also described by which c‐Myc promoted transcription of purine biosynthetic enzymes by direct binding to the promoter region [111].

## 5 Therapeutic approaches exploiting signaling and metabolism in GBM

### 5.1 Metabolic and signaling inhibitors to target GBM

**Fig. 6:**
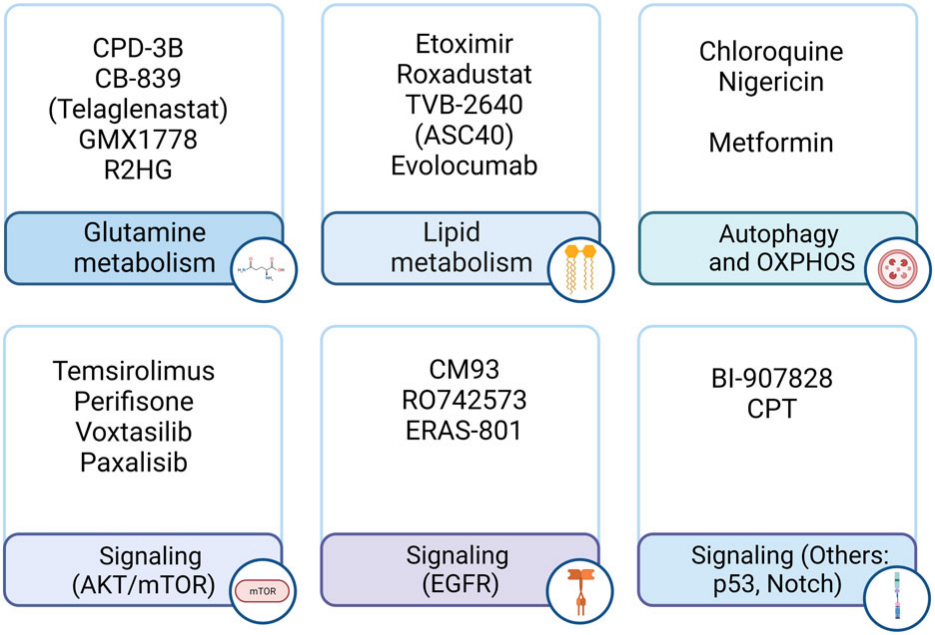
Metabolic and signaling‐targeting drugs on clinical trials for GBM. Created with Biorender.com.

Several glutaminolytic and glycolytic enzymes are potential therapeutic targets for blocking the main sources of energy, carbon and nitrogen for GBM. A compensatory metabolism developed in IDH‐mutant gliomas to sustain glutamate levels leads to a dependency on glutamine metabolism. The oncometabolite d‐2‐hydroxyglutarate produced by mutated IDH, directly inhibits BCAT transaminases reducing branched‐chain amino acid catabolism and consequently glutamate levels [112]. The study shows that BCAT inhibition by the R‐enantiomer of 2‐hydroxylglutarate (R2HG) induces a compensatory increase in glutamine metabolism to sustain glutamate that leads to a dependency on GLS activity. Indeed, treatment of orthotopic xenograft GBM tumors with GLS inhibitors, including the most potent allosteric GLS inhibitor CB‐839, specifically sensitizes IDH‐mutant gliomas and not IDH‐wild type gliomas to radiation treatment [112]. This drug (also known as Telaglenastat) is currently part of a phase I clinical trial to test dose and side effect in low‐grade astrocytomas IDH‐mutant, in combination with radiotherapy and TMZ (NCT03528642) (Fig. 6).

New specific GLS inhibitors have been described. The dual inhibitor of GLS and GDH, CPD‐3B (Hexylselen) has demonstrated to effectively disrupt mitochondrial membrane potential and to induce apoptosis of glutamine addicted cancer cells without showing toxicity to normal cells [113]. In a preclinical study of a mouse hepatocellular carcinoma xenograft model, this dual inhibitor of glutaminolysis showed a significant efficacy with reduced tumor size, increased tumor necrotic area and prolonged survival rate [114]. Whether CPD‐3B could have a relevant effect in GBM tumors with enhanced glutaminolysis is still unknown and no clinical trial is testing this drug yet.

The rate‐limiting enzyme nicotinamide phosphoribosyltransferase (NAMPT) which regulates NAD+ levels, has been revealed as a potential druggable target against Myc‐driven GBM cells. The deleterious effect of NAMPT inhibitors seems to be due to the depletion of NAD+ levels, necessary to sustain the Myc‐mediated increase in glycolysis flux [78]. The preclinical studies with orthotopic GBM xenografts have shown an increase in mouse survival with increased tumor cell death after oral administration of the NAMPT inhibitor GMX1778 [78]. No clinical trials have been developed to test this inhibitor in GBM.

Recent studies have highlighted the dependency of certain types of gliomas to the *de novo* pyrimidine biosynthesis with potential druggable purposes. These studies show that the highly aggressive pediatric diffuse midline gliomas and the adult IDH‐mutant gliomas are particularly vulnerable to pyrimidine synthesis inhibition. A brain penetrant DHODH inhibitor, BAY2402234, was tested in both glioma models. BAY2402234 has shown effectiveness in prolonging survival of mice bearing intracranial diffuse midline glioma xenografts, reducing *in vivo* levels of DHODH downstream metabolites [115]. Similarly, BAY2402234 treatment reduces *in vivo* tumor growth and, in some cases, displays tumor regression in mice bearing IDH‐mutant glioma allografts [107]. BAY2402234 recently entered in an early‐phase I clinical trial (NCT05061251) for recurrent glioma that was subsequently withdrawn due to drug availability.

The deleterious effect of Notch inhibition in GBM and GSCs integrity has been widely documented. The most employed therapeutic approach targeting Notch signaling in cancer are γ‐secretase inhibitors (GSIs) which prevent the release of the Notch intracellular domain and therefore signaling activation. GSIs have been tested in clinical trials for GBM either primary or recurrent. However due to side effects, toxicity and lack of Notch receptor specificity, other approaches are currently being considered, including receptor‐ or ligand‐specific humanized antibodies and compounds targeting downstream components of the pathway. In this regard, the increasing data linking Notch signaling with metabolic regulation and therapy resistance mechanisms could be exploited against GBM. As previously described for the Myc pathway, studies toward understanding Notch‐associated metabolic phenotypes could show novel vulnerabilities for future therapeutic intervention [75]. The interaction of Notch2 with mitochondrial complex I to sustain reverse electron transfer has shown to affect NAD+ metabolism, and more specifically the NAD+/NADH balance. Indeed, the inhibition of either Notch2/complex I interaction with the quinazolinone‐based drug CPT, or NAMPT, results in a similar deleterious effect on GBM cell viability [75]. This same study showed that CPT has anticancer activity in a preclinical study using orthotopic GBM xenografts [75]. This novel small molecule compound is under patent and there are no clinical trials testing this drug yet. Whether NAMPT inhibitors could be effective in GBM with hyperactive Notch should be also considered. Novel approaches could also take advantage of a recent study showing crosstalk between Notch2 and *N*‐acetylcysteine (NAC) [116]. NAC treatment attenuates Notch2 levels and signaling by promoting its lysosomal degradation which leads to the inhibition of GBM cell proliferation and reduced tumor growth *in vivo*. Of note, NAC is already being tested in several ongoing clinical trials for other solid tumors including ovarian cancer (NCT04520139) and lymphoma (NCT05081479) but not for GBM.

In human patient‐derived glioma cells, the antibiotic nigericin suppresses cancer stem cell properties through a mechanism involving AMPK phosphorylation and mTORC1 inactivation leading to autophagy induction. Furthermore, malignant characteristics of human glioma cells were markedly suppressed by nigericin treatment *in vivo* [117]. Targeting of autophagy is also evaluated in GBM using the autophagy inhibitor chloroquine alone or in combination with radio and chemotherapy [118].

Metabolic targeting has also emerged to overcome therapy‐mediated metabolic compensatory reprogramming. A study with U87 cells and GBM xenografts has shown that GLS inhibition sensitizes to mTOR inhibitors by blocking the enhanced glutamine metabolism induced as a compensatory reaction against mTOR inhibition [66]. The resistance of GBM to PI3K and AKT inhibitors relies at least partially on the compensatory activation of an mTORC2/c‐Myc pathway that leads to enhanced glycolysis and cell survival. The development of the dual inhibitor XL765 (Voxtalisib) targeting both PI3K and mTOR pathways to block the compensatory response has shown a potent reduction of tumor growth in a patient‐derived GBM xenograft model [83]. Two clinical trials were conducted and completed using XL765 alone (NCT01240460) or in combination with TMZ (NCT00704080) in recurrent or primary adult GBM with no results presented, which suggests that despite the promising preclinical studies, this therapeutical approach did not clinically succeed.

It is known that IDH‐mutant GBM tumors are dependent on NAD+ for survival, representing a genotype‐specific vulnerability in IDH‐mutant gliomas. Base excision repair, activated by TMZ‐induced DNA damage, relies on NAD+ availability after PARP activation. The combination of TMZ with NAMPT inhibitors, which blocks NAD+ biosynthesis, improved TMZ efficacy in an *in vivo* IDH1‐mutant GBM model [119].

### 5.2 Clinical trials

More than 500 clinical studies are currently in the recruitment stage or already active for evaluation, for both newly diagnosed and recurrent GBM. These studies include a wide variety of signaling‐, metabolic‐ or DNA and proteome stability‐related drugs (Table 2). Current clinical trials for GBM targeting signaling pathways include a phase I trial studying the effect of perifosine and temsirolimus for recurrent malignant gliomas (NCT02238496) (Fig. 6). Given the overactive PI3K/AKT/mTOR axis in GBM, the preclinical study for the dual inhibition of AKT with perifisone and mTOR with temsirolimus, has already shown synergistic anti‐tumor effects [120]. Other clinical trials targeting EGFR are currently active using different EGFR inhibitors including CM93, a potent EGFR inhibitor with positive results in recurrent GBM with mutant‐EGFR (NCT04933422) [121]; RO7428731 is also in phase I for newly diagnosed and recurrent GBM positive for EGFRvIII (NCT05187624); or ERAS‐801, a CNS‐penetrant EGFR/ERBB1 inhibitor, is in a phase I study for recurrent GBM (NCT05222802). Several other ongoing trials are focused on neutralizing p53 regulators such as MDM2 inhibitor BI‐907828 (NCT05376800). BI‐907828 has shown promising results in *TP53* wild‐type GBM by decreasing viability in *in vitro* and *in vivo* studies, and enhancing survival in orthotopic xenograft mouse models [122]. At the moment there are no active clinical trials targeting Notch signaling. Previous trials were mainly focused on GSIs but they were completed with no beneficial outcome or terminated prematurely by drug suppliers (i.e., NCT01122901, NCT01119599, NCT01269411).

**Table 2:** Ongoing clinical trials targeting signaling and metabolic elements in GBM. NTC: number of ClinicalTrials.gov identifier.

NTC number	Target	Drug	Phase	Title
NCT04933422	EGFR	CM93	1	CM93 Treatment in Subjects With Epidermal Growth Factor Receptor (EGFR)‐Modified Recurrent Glioblastoma (rGBM)
NCT05187624	EGFRvIII	RO7428731	1	A Study Evaluating the Safety, Pharmacokinetic and Anti‐tumor Activity of RO7428731 in Participants With Glioblastoma
NCT05222802	EGFR/ERBB1	ERAS‐801	1	A Study to Evaluate ERAS‐801 in Patients With Recurrent Glioblastoma (THUNDERBBOLT‐1)
NCT05376800	p53	BI‐907828	1	A Study to Determine How BI 907828 is Taken up in the Tumor and to Determine the Highest Dose of BI 907828 That Could be Tolerated in Combination With Radiation Therapy in People With a Brain Tumor Called Glioblastoma
NCT02238496	AKT/mTOR	Perifosine and temsirolimus	1	Perifosine and Torisel (Temsirolimus) for Recurrent/Progressive Malignant Gliomas
NCT05183204	PI3K/mTOR and OXPHOS	Paxalisib with metformin	2	Paxalisib With a High Fat, Low Carb Diet and Metformin for Glioblastoma
NCT04945148	OXPHOS	Metformin, TMZ and radiation	2	Oxidative Phosphorylation Targeting In Malignant Glioma Using Metformin Plus Radiotherapy Temozolomide (OPTIMUM)
NCT04945148	OXPHOS	Metformin	2	Oxidative Phosphorylation Targeting In Malignant Glioma Using Metformin Plus Radiotherapy Temozolomide (OPTIMUM)
NCT03528642	Glutaminase (glutamine metabolism)	Telaglenastat	1	Telaglenastat With Radiation Therapy and Temozolomide in Treating Patients With IDH‐Mutated Diffuse Astrocytoma or Anaplastic Astrocytoma
NCT02715609	Aldehyde dehydrogenases (ALDHs)	Disulfiram/Copper	1, 2	Disulfiram/Copper With Concurrent Radiation Therapy and Temozolomide in Patients With Newly Diagnosed Glioblastoma
NCT04990739	ACSS2 (acetate metabolism)	MTB‐9655	1	Study of MTB‐9655, an Inhibitor of ACSS2, in Patients With Advanced Solid Tumors
NCT03032484	FASN (lipid metabolism)	TVB‐2640 and bevacizumab	2	TVB‐2640 in Combination With Bevacizumab in Patients With First Relapse of High Grade Astrocytoma
NCT05118776	FASN (lipid metabolism)	TVB‐2640 and bevacizumab	3	Study to Evaluate the Safety and Efficacy of ASC40 Tablets in Combination With Bevacizumab in Subjects With rGBM
NCT04937413	PCSK9 (lipid metabolism)	Evolocumab	1	The PCSK9i Inhibitor Evolocumab – a Surgical Trial of Pharamcodynamics and Kinetics Evaluation
NCT04250922	SGSM1 (lipid metabolism)	2‐OHOA	2, 3	2‐OHOA With RT and TMZ for Adults With Glioblastoma (CLINGLIO)
NCT02432417	Autophagy	Chloroquine	2	The Addition of Chloroquine to Chemoradiation for Glioblastoma

A glutamine dependency in GBM tumors has led to the development of therapeutic approaches targeting tumor metabolism (Table 2). A phase I trial combines the glutaminase inhibitor telaglenastat with radiation and TMZ in IDH1‐mutant astrocytomas (NCT03528642). Lipid metabolism is also important in GBM progression and given the implications of FASN in GBM tumorigenesis, therapeutic agents targeting FASN are being tested. A phase II trial using the FASN inhibitor TVB‐2640 (also known as ASC40) in combination with the anti‐angiogenic antibody bevacizumab in recurrent GBM has shown optimistic results recently (NCT03032484). The progression‐free survival at 6 months (PFS6) observed for TVB‐2640 plus bevacizumab was 47%, representing a statistically significant improvement over historical bevacizumab monotherapy. Based on this phase II study, a phase III is now on recruitment status to compare the safety and effectiveness of the ASC40 tablets in combination with bevacizumab against bevacizumab alone (NCT05118776). Another clinical trial targeting lipid metabolism is an early‐phase I to evaluate the pharmacokinetics and pharmacodynamics of PCSK9 inhibitor evolocumab in primary and recurrent GBM (NCT04937413). PCSK9 is a proprotein convertase affecting cholesterol levels by regulating the amount of low‐density lipoprotein receptors at the cell membrane [123]. The primary aim of this trial is to evaluate whether evolocumab crosses the blood–brain barrier and is detectable in the resected tumor specimens of patients with primary and recurrent GBM.

OXPHOS inhibition is also being evaluated for GBM treatment. A phase II trial for newly diagnosed IDH‐wild type GBM particularly dependent on OXPHOS is opened although not yet recruiting (NCT04945148). The treatment consists of the combination of metformin, an oral inhibitor of mitochondrial complex I, with radiotherapy and TMZ. In another already recruiting phase II trial it is evaluated the safety and effect of dual inhibition of OXPHOS and PI3K/mTOR signaling in newly diagnosed and recurrent GBM. This study combines the PI3K/mTOR inhibitor paxalisib with metformin while maintaining a ketogenic diet (high fat/low carbohydrate diet) (NCT05183204).

The preclinical studies targeting acetate metabolism by ACSS2 inhibition point to ACSS2 as a potentially druggable target in GBM therapy development [64, 87]. A first‐in‐class small molecule inhibitor of ACSS2, MTB‐9655, has been already developed and is currently in a phase I clinical trial to establish a safe and tolerable dose in patients with advanced or metastatic solid tumors for which standard therapy has failed (NCT04990739). Although no information is available regarding the types of tumors enrolled in this study, GBM could fulfill the inclusion criteria.

## 6 Concluding remarks

Alterations in signaling pathways or reprogramming of cellular metabolism introduces new promising opportunities for GBM treatment. Targeting these alterations is an alternative that may provide some benefit in this challenging disease. However, to date, and despite all the clinical trials testing the possibility of targeting metabolic transformation to attack GBM tumors in patients, this possibility is still far from being a reality. Our knowledge about the relationship between all the molecular drivers, essential for the design of combined therapies, is still very limited. Future work would aid to increase the efficiency of the current standard of care of GBM, TMZ, by the integration of the research result of the last decades.

## Conflict of interest

The authors declare no conflict of interest.

## Author contributions

LZ, RVD and MT wrote and revised the manuscript.
